# *Things seen and* unseen: 2. Anaemia affects urban rich Nigerian adolescents more than other socio‑economic status groups

**DOI:** 10.5334/aogh.4551

**Published:** 2024-11-04

**Authors:** Chukwunonso ECC Ejike, Nkechi Igwe‑Ogbonna, Nneoma Uwadoka

**Affiliations:** 1 Alex Ekwueme Federal University, Ndufu‑Alike, Ebonyi State, Nigeria

**Keywords:** Adolescence, anaemia, Nigeria, prevalence, socio‑economic status

## Abstract

*Background:* Anaemia is very prevalent globally and is thought to be linearly associated with wealth and to affect females and rural residents more than males and urban residents.

*Objectives:* This study was designed to investigate this thought in a population of adolescents in Ebonyi State, Nigeria.

*Methods:* Standard clinical protocols were used. A total of 362 adolescents (63.5% females) were studied. Anaemia was diagnosed on the basis of the World Health Organization (WHO) criteria.

*Results:* Aggregate anaemia was found in 50.0% of the general population (43.9% males, 53.5% females) and was most prevalent in the urban upper socio‑economic status (SES) group (62.3%; 70.0% females, 52.2% males). Severe anaemia was present in 7.2% of the general population (9.1% males, 6.1% females). It was most prevalent amongst the 10–11 years age bracket (15.6%). Moderate and mild anaemia were found in 22.4% and 20.4% of the general population and in 13.0%, 11.4%, 8.3% and 6.0% of the urban upper, rural, urban low and middle SES groups, respectively. But in the rural area group, severe anaemia existed without wide sex variations. Moderate anaemia was most prevalent in the urban middle and upper SES groups (29.0% and 26.4%, respectively) with a clear female preponderance. Mild anaemia was the least prevalent (15.3%) in the urban middle SES group.

*Conclusions:* The higher prevalence of severe anaemia in boys and the higher burden in the urban higher SES group warrant a rethink of the public health interventions used in Nigeria. Adolescent boys and urban upper SES groups should be targeted in nutrition interventions related to anaemia.

## Introduction

Anaemia is described as a medical condition characterised by haemoglobin concentrations being below the recommended threshold needed to support normal metabolic functions. This results in insufficient oxygen carrying capacity of the blood and the attendant poor oxygen delivery to tissues [1, 2]. Anaemia was estimated to affect approximately 1.8 billion individuals globally in 2019, accounting for nearly 50.3 million years lived with disability. In the 30 years between 1990 and 2019, the prevalence of anaemia was reduced by 13.4% globally [3]. It is reported that anaemia is more prevalent in low‑ and middle‑income countries (LMICs) and that sub‑Saharan Africa bears a disproportionate burden of the disease [4]. In Nigeria, prevalence values of 71% have been reported for children under 5 years of age [5] and 32% for those aged 5–12 years [6].

Anaemia is caused by a variety of factors. However, half of all anaemia cases are reported to be due to iron deficiency anaemia (IDA) [7]. The other half are attributable to genetic disorders such as haemoglobinopathies and thalessaemias, infections such as malaria and human immunodeficiency virus (HIV), infestations such as intestinal parasites, chronic diseases associated with inflammation such as obesity and rheumatoid arthritis, and deficiencies in nutritional factors such as folate and vitamin A [8]. Anaemia is associated with poor cognitive development and school performance [9], reduced immunity and infection resistance, poor physical growth outcomes, physical fitness, reduced work capacity, and generally increased mortality [10]. It is also associated with impaired thermoregulation, tuberculosis and heart failure [11].

There are reports that anaemia is linearly associated with wealth [12, 13] and that females and rural residents are more affected than males and urban residents [14, 15]. Again, anaemia is reported to be preponderant in children under 5 years old [16], and infants are known to rely on maternal iron endowment for the first few months of life (as breast milk is deficient in iron) [17]. These have resulted in research and public health efforts related to anaemia targeting the poor, especially in rural areas, women of child‑bearing age/pregnant women and the girl‑child. Interestingly, however, there are reports that urban‑poor children and adolescents are more adversely affected by malnutrition compared with their rural counterparts [18] and that male infants and young boys have lower iron stores relative to their female counterparts and thus a raised risk of iron deficiency anaemia [16, 19].

Adolescence is that transitional stage of life before adulthood. It is characterised by increased growth and development and is the last opportunity for catch‑up growth and ‘make‑up’ for any nutritional deficiencies before the onset of adulthood [18]. Adolescent girls (in most parts of sub‑Saharan Africa) are often a few years away from getting married and becoming mothers, with all the attendant biological demands associated with it. Pregnant women, if anaemic, face the risk of maternal morbidity and mortality, premature delivery, delivery of low birth weight infants and perinatal mortality. Indeed, infants born to anaemic mothers are known to have a greater risk of anaemia in the first 6 months of life [20]. Unfortunately, adolescents often do not show overt signs of ill‑health, thereby giving the false impression of being healthy. The consequence is the reduced research attention paid to this age‑bracket [18]. Additionally, studies on anaemia have not paid sufficient attention to the effect of socio‑economic status (SES) on its dynamics within the same geographical area. This study was therefore designed to investigate the prevalence of anaemia in a population of adolescents aged 10–18 years in Ebonyi State, Nigeria and to explain the influence of SES on its epidemiology.

## Subjects and Methods

### Location and subjects

**Figure 1:**
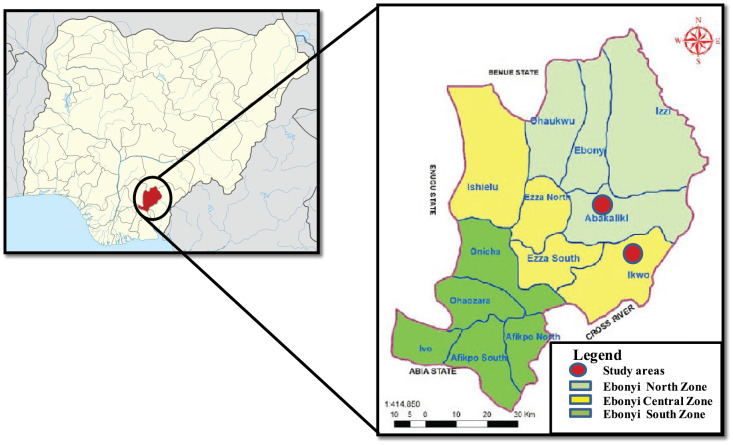
Map of Ebonyi State showing the study areas.

This was a cross‑sectional study of adolescents in Ebonyi State, Nigeria. Ebonyi is one of the five states in the South‑East geo‑political zone of Nigeria, predominantly populated by the Igbo ethnic group. It is the poorest state in the zone and the fourth poorest in Nigeria [21]. School‑going children and adolescents were recruited from selected secondary schools in Abakaliki and Ikwo. Abakaliki is the capital of Ebonyi State and an urban city. Ikwo is a rural agrarian local government area (LGA) in the state. Though Abakaliki and Ikwo are in two different geopolitical areas of the state, both LGAs are contiguous (Figure 1). Schools were purposely selected from the school’s list, and the principals of the schools were written formally, seeking permission to conduct the study in their schools. Fees charged by the schools were used as a proxy for SES in the urban area. Consequently, schools where tuition is free or less than ₦60,000.00 (∽$40) per annum were chosen to represent urban low SES, those that charge ₦90,000–₦150,000 (∽$60–$100) per annum were chosen to represent urban middle SES, while those that charge more than ₦300,000 (∽$200) per annum were chosen to represent the urban upper SES.

A total of nine schools (56.3% of those written to), four schools in the rural area and five others in the urban area, for which their principals gave written consent, were chosen for the study. Letters were written to the parents/guardians of the students, informing them of the objectives of the study and methods to be used, and seeking their consent to allow their children/wards to participate in the study. Only students whose parents/guardians gave written consent were allowed to participate in the study. Students who had any overt signs of ill‑health, reported suffering from malaria or any infection/infestation in the 2 weeks prior to the study or receiving treatment for the same at the time of the study, or reported being cigarette smokers, were excluded from the study. A total of 362 subjects (63.5% females) were effectively recruited for the study and distributed as follows: rural dwellers, 86 subjects (59.3% females); urban low SES, 99 subjects (75.8% females); urban middle SES, 124 subjects (59.7% females); and urban upper SES, 53 subjects (56.6% females). None of the participants had sickle cell disease and none of the females were pregnant.

### Methods

Self‑reported date of birth was recorded for each subject and their ages were determined. Using a structured questionnaire, information was obtained on who each subject lived with, their means of transportation to school and whether or not they went to school with snacks. Haemoglobin concentration for each subject was determined using a portable haemoglobinometer, HemoCue Hb 301 (HemoCue AB, Angelholm, Sweden). Capillary blood samples were aseptically collected from each subject’s fingertip and placed on the test strip which takes up the blood by capillary action. The concentration of haemoglobin in the blood sample is determined by photometric estimation using the cyanmethaemoglobin method [22] and is digitally displayed in less than a minute. Haematocrit was automatically calculated by the device by multiplying the red cell number by the mean cell volume (MCV), and the output was displayed as percentage of blood volume.

Anaemia was diagnosed and classified on the basis of the recommendations of the World Health Organization as follows: severe anaemia (haemoglobin concentration < 80 mg/L), moderate anaemia (haemoglobin concentration of 80–109 mg/L), and mild anaemia [haemoglobin concentrations of (i) 110–114 mg/dL in children aged 5–11 years, (ii) 110–119 mg/dL in children aged 12–14 and in non‑pregnant females older than 15 years, and (iii) 110–129 mg/dL in males older than 15 years] [1]. Subjects with severe, moderate or mild anaemia were grouped as having aggregate or total anaemia.

The study protocol was prepared in accordance with the Helsinki Declaration. Ethical approval for the study was obtained from the Board of the Department of Biochemistry, Alex Ekwueme Federal University, Ndufu‑Alike, Ebonyi State. Participants were given tips on healthy living and a lecture on anaemia before the study. Those that were diagnosed with anaemia were informed and appropriately advised, privately. No honoraria were paid to the participants. However, stationery was given to participants as tokens of appreciation.

### Data analysis

The data generated was subjected to basic descriptive statistical analysis and were reported as mean ± standard deviation for continuous data and percentages for categorical data. Differences between means for continuous data were separated by one‑way analysis of variance (ANOVA) and a significant threshold of *P* < 0.05 was adopted. The results are presented in tables of figures generated using Microsoft Office applications. Due to the small sample sizes, the data were grouped as per the age ranges used in the WHO diagnostic criteria (WHO 2011), where necessary.

## Results

A total of 91.9% of the subjects from the urban middle and upper SES groups live with their parents, compared with 46.5% of those in the urban low SES group (Table 1). In the rural group, the figure was 76.7%. While 70.9% and 83.8% of the rural and urban low SES groups, respectively, walked to school daily, only 18.5% and 13.2% of the urban middle and upper SES groups, respectively, went to school in a vehicle, daily. Amongst the urban low SES students, 26.3% reported going to school with snacks, while 54.7–60.5% of their counterparts in the other SES groups went to school with snacks daily.

**Table 1:** Sociodemographic characteristics of the subjects, stratified by socio‑economic status.

		PROPORTION OF THE POPULATION THAT		
		LIVES WITH PARENTS (%)	WALKS TO SCHOOL DAILY (%)	GOES TO SCHOOL WITH SNACKS (%)
**Rural**	Female (*N* = 51)	74.5	72.5	64.7
	Male (*N* = 35)	80.0	68.6	40.0
	Total (86)	76.7	70.9	54.7
**Urban low SES**	Female (*N* = 75)	44.0	85.3	30.7
	Male (*N* = 24)	54.2	79.2	12.5
	Total (99)	46.5	83.8	26.3
**Urban middle SES**	Female (*N* = 74)	91.9	18.9	68.9
	Male (*N* = 50)	92.0	18.0	48.0
	Total (124)	91.9	18.5	60.5
**Urban upper SES**	Female (*N* = 30)	93.3	6.7	53.3
	Male (*N* = 23)	79.2	20.8	54.2
	Total (53)	88.7	13.2	54.7

Rural dwellers and urban low SES groups were slightly older than urban middle and upper SES groups. In the rural and urban upper SES groups, the mean ages of the males and females were statistically similar (*P* > 0.05), while the males in the urban low and middle SES groups were significantly (*P* < 0.05) older than their female counterparts. Other than the rural group, mean haemoglobin concentrations and haematocrit values were marginally higher in the males compared with the females. The differences were, however, significant (*P* < 0.05) only in the urban middle SES group (Table 2).

**Table 2:** Mean age, haemoglobin and haematocrit values of subjects stratified by socio‑economic status.

		AGE (YEARS)	HEAMOGLOBIN CONCENTRATION (G/L)	HEAMATOCRIT (%)
**Rural**	Female (*N* = 51)	14 ± 2	120.1 ± 25.5	35 ± 4
	Male (*N* = 35)	15 ± 2	116.2 ± 27.5	34 ± 8
	*P*	0.162	0.382	0.389
**Urban low SES**	Female (*N* = 75)	15 ± 2	116.9 ± 23.8	34 ± 7
	Male (*N* = 24)	16 ± 2	119.3 ± 20.9	35 ± 6
	*P*	0.003	0.663	0.721
**Urban middle SES**	Female (*N* = 74)	13 ± 2	112.3 ± 20.2	33 ± 6
	Male (*N* = 50)	14 ± 2	122.1 ± 22.2	36 ± 7
	*P*	0.023	0.012	0.031
**Urban upper SES**	Female (*N* = 30)	12 ± 2	107.4 ± 19.5	31 ± 6
	Male (*N* = 23)	12 ± 2	113.7 ± 27.3	32 ± 8
	*P*	0.118	0.329	0.598

**Figure 2:**
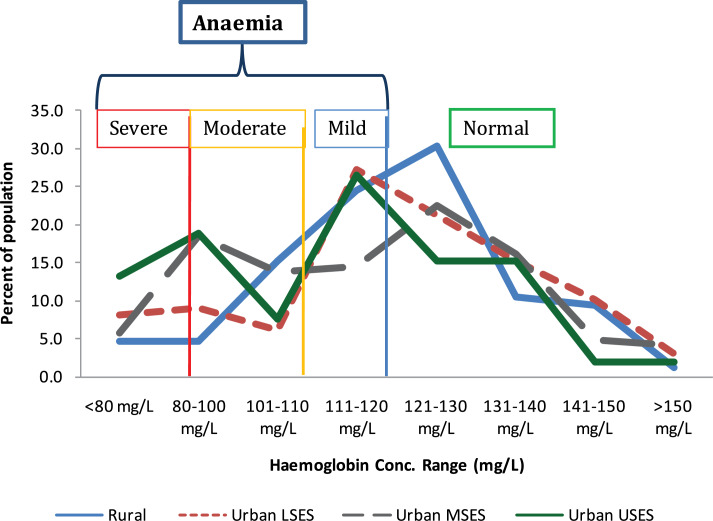
Distribution of haemoglobin concentrations in the population.

**Figure 3:**
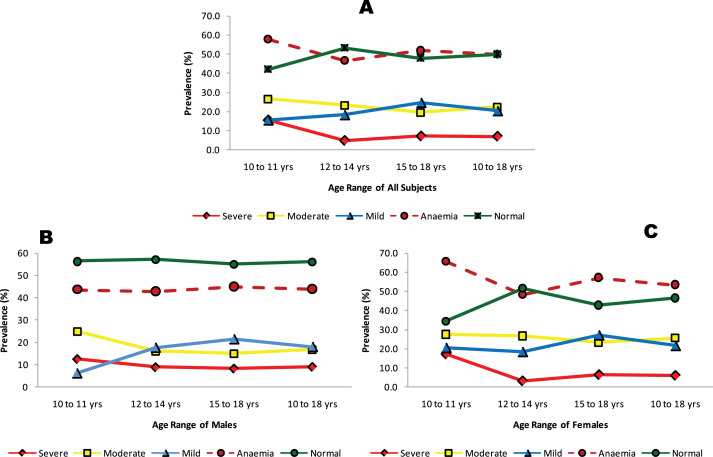
Prevalence of anaemia in the population stratified by sex and age. **3A**, all subjects irrespective of sex; **3B**, male subjects; **3C**, female subjects.

From Figure 2 it is seen that the majority of the subjects, irrespective of their socio‑economic status, had haemoglobin concentrations that predominantly fell into the mild anaemia or normal part of the spectrum. Aggregate or total anaemia was found in 50.0% of the studied population (43.9% in males and 53.5% in females) (Figure 3). It was most prevalent among 10–11‑year‑old subjects (57.8%), especially the females (65.5%), and was least prevalent amongst 12–14 year olds (46.7%), especially the males (42.9%).

Severe anaemia was present in 7.2% of the population (9.1% males and 6.1% females). It was most prevalent amongst the 10–11 years age bracket [15.6% (12.5% males, 17.2% females)] and least prevalent amongst the 12–14 years age bracket (5.0%), particularly the females (3.3%). Moderate anaemia was found in 22.4% of the population (16.7% for males and 25.7% for females). The prevalence of moderate anaemia decreased with age (albeit modestly) irrespective of sex from 26.7% amongst 10–11 year olds to 19.7% amongst 15–18 year olds. The least prevalence recorded for moderate anaemia was amongst 15–18‑year‑old males (15.0%). Mild anaemia prevalence was 20.4% (18.2% for males and 21.7% for females). Mild anaemia increased with age in the general population (Figure 3A) and was highest amongst 15–18‑year‑old females (27.3%) (Figure 3C) and lowest amongst 10–11‑year‑old males (6.3%) (Figure 3B).

**Figure 4:**
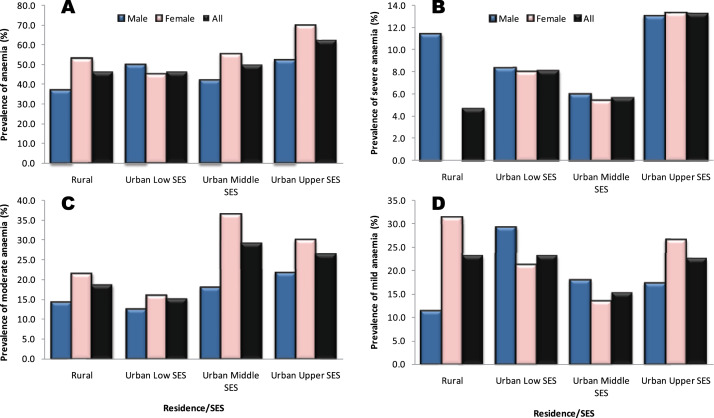
Prevalence of anaemia in the population stratified sex and socioeconomic status. **4A**, total anaemia; **4B**, severe anaemia; **4C**, moderate anaemia; **4D**, mild anaemia.

From Figure 4, it is seen that aggregate or total anaemia was most prevalent in the urban upper SES group (62.3%) where more females than males were affected (70.0% versus 52.2%). It was the least prevalent in the urban middle SES group, irrespective of sex (50%), but amongst rural males (37.1%) (Figure 4A). Severe anaemia was found in 13.0% of the urban upper SES group (irrespective of sex). The figures for the urban low and middle SES groups were 8.3% and 6.0%, respectively, without significant sex variations. In the rural area, however, 11.4% males and no females had severe anaemia (Figure 4B).

Moderate anaemia was most prevalent in the urban middle and upper SES groups (29.0% and 26.4%, respectively) with a clear female preponderance (Figure 4C). The prevalence of moderate anaemia was least amongst the urban low SES group, irrespective of sex. Mild anaemia was found in 23.3%, 23.2% and 22.6% of subjects in rural, urban low and urban upper SES groups, respectively, and 15.3% of the urban middle SES group (Figure 4D). However, the prevalence was highest amongst urban low SES males (29.2%) and rural females (31.4%).

## Discussion

The prevalence of anaemia (severe, moderate and mild) found in this study was 50.0%, but 29.6% if mild anaemia was discountenanced. A meta‑analysis of data from Ethiopia gave a prevalence of 34.4% for children and adolescents younger than 14 years [23]. A more recent meta‑analysis, also in Ethiopia, but amongst 15–19‑year‑old girls gave a prevalence of 23.0% for anaemia [24]. A trend analysis using data from the Demographic and Health Surveys (DHS) spanning the years 2000–2018 for girls aged 15–19 years in sub‑Saharan Africa reported a prevalence of anaemia of 36.0% in the region [13]. In Bangladesh, a recent systematic review and meta‑analysis of studies reported a pooled prevalence of 42.1% (28.5–55.7%) for anaemia in 10–19‑year‑old adolescents, after examining nine studies that enrolled a total of 6,267 subjects [25]. They also reported a pooled prevalence of 53.5% (26.2–80.8%) for non‑severe anaemia from four studies that enroled a total of 2,390 subjects in the same age‑bracket. Though anaemia was determined by haemoglobin measurements by the different authors whose works were used for the reviews cited above, the differences in categorisations/definitions used make direct comparisons difficult. Furthermore, geographical differences are known to cause 34–43% of observed variations in the prevalence of anaemia [26]. What is nonetheless clear is that the prevalence of aggregate or total anaemia in the studied population is high and is either higher or comparable to figures from other tropical low and middle income countries. The concordance between the values for haemoglobin and haematocrit found in this study is expected, as the latter is derived from the former.

The WHO prescribes that anaemia (determined by haemoglobin measurements) in a population is considered a severe, moderate or mild public health importance if the prevalence is ≥ 40%, 20.0–39.9% or 5.0–19.9%, respectively [1]. Therefore, aggregate or total anaemia, at more than 40% prevalence in each of the sexes, is of severe public health importance based on the WHO categorisation. However, when anaemia is disaggregated, it is seen that severe anaemia (in both sexes and irrespective of age and SES) in the population (based on the same categorisation) is of mild public health importance, while moderate and mild anaemia, also irrespective of sex, age and SES, are each of moderate public health importance. In Nigeria and most parts of sub‑Saharan Africa, the high prevalence of infectious diseases such as malaria, infestations such as helminthiasis, haemoglobinopathies (such as sickle cell anaemia) and micronutrient deficiencies (such as deficiencies of iron, cobalamin, folate and vitamin A) are reported to be responsible for anaemia in as much as 47% of children and adolescents [27]. Besides these, it is known that the most common cause of anaemia globally is iron deficiency. This is usually a consequence of inadequate intake of iron‑rich foods, especially for adolescents (and pregnant women) that have high demands for nutrients for growth and metabolism. The prevalence of anaemia reported in this study warrants urgent public health action as it is indicative of the slow progress being made by current interventions.

Severe and moderate anaemia was more prevalent in 10–11‑year‑old boys and girls, while mild anaemia was more prevalent in the 15–18‑year‑old adolescents. This is in consonance with reports that adolescent girls in their early adolescence were more likely to be anaemic than adolescents in their later age [14]. Early adolescence appears to be an age bracket requiring particular attention with respect to severe anaemia. Severe anaemia was more preponderant in boys than girls, while moderate and mild anaemia was more prevalent in girls than boys. This discordance is as noteworthy as the finding that boys were affected more by severe anaemia than girls. Indeed, in the rural setting, we found no girl with severe anaemia. What is more commonly reported in the literature is the preponderance of anaemia in girls, irrespective of age [15, 16, 28]. This has been attributed to onset of menstruation in younger adolescents and heavy menstruation in older ones [15]. Additionally, haemoglobin concentrations are positively and inversely associated with skeletal muscle mass and fat mass, respectively, directly, and with physical fitness, indirectly [29, 30]. This is thought to place the boys (who have more skeletal muscles than the girls and who are often more physically fit due to cultural practices that reserve jobs requiring physical exertion for boys) at a haemoglobin advantage.

Our findings, however, agree with reports by Safiri *et al.* [3] who found that anaemia was more prevalent in male children compared with their female counterparts. This observation had been reported by earlier studies [19, 31] and a review [16]. It is thought that the effects of hormones (especially testosterone) on erythropoietin activity coupled with the higher pre‑ and post‑natal growth rate found in boys may play a role in the observed prevalence of severe anaemia in male children [31]. Furthermore, it is plausible that reverse causation may explain the relationship between anaemia in boys and girls. It is possible that public health messages have consistently targeted female children, especially during ante‑natal education of mothers, such that mothers now preferentially provide their daughters with iron‑rich foods and/or supplements at the expense of their sons. Public health interventions in Nigeria and elsewhere such as iron/folate supplementation and de‑worming target adolescent girls but not boys [32]. The chickens may have come home to roost for such policies. More studies are warranted to unravel the reasons for the discordance in the prevalence of severe and moderate/mild anaemia with respect to the sexes.

Severe anaemia was more prevalent in urban upper SES boys and girls and rural boys. Moderate anaemia was more prevalent in urban upper and middle SES groups with a clear female preponderance. Mild anaemia was equally prevalent in rural, urban upper and low SES groups, and lowest in the urban middle SES group. Clearly, the urban upper SES group had an unexpected disproportionate share of the anaemia burden. This is quite contrary to popular thought on the relationship between anaemia and SES, which dictates that anaemia is associated with lower SES and rural residence [12, 13, 15, 33, 34]. The explanation given by such studies for this relationship are usually poverty and lack of access to micronutrient‑rich foods, high prevalence of infections and infestations, poor hygiene conditions, low literacy and access to useful health information, etc.

Our data, however, suggest that different pathways may be at play. It appears that the advantages conferred on the higher SES class and urban residents may be exaggerated, at least in the studied population. Whereas children of higher SES groups in Nigeria are consistently reported to have better indices of (macro‑) nutritional status [18, 35, 36], macro‑nutrient sufficiency does not imply micro‑nutrient sufficiency. It is possible that the urban rich have useful health information but have not put it to use. There are indeed reports of low dietary iron intakes and high prevalence of iron deficiency in both low and higher income geographical locations [8]. Urban areas also often have more breeding grounds for disease vectors such as mosquitoes [37], which may increase the prevalence of infectious diseases such as malaria.

Beyond the above, it is plausible that reverse causation (again) may explain the observed higher prevalence of anaemia in boys and girls of upper SES. Because public health messages consistently highlight the higher burden of anaemia in the poor and rural dwellers, the groups have been targeted, and the urban rich are lost in a presumed freedom from anaemia. Here, it is important to note that factors that increase anaemia such as elevated production of pro‑inflammatory cytokines and free radicals, which damage erythroid progenitor cells, and cardiometabolic risk biomarkers (such as hypercholesterolaemia and impaired glucose homoeostasis), both of which being markers of adiposity and the attendant inflammatory response [38, 39], are more prevalent amongst the higher SES group. This may also explain some of our findings.

Furthermore, physical fitness and exercise elevates haemoglobin concentration [30]. The data on living with parents, mode of transportation to school and taking a snack to school validate our use of fees as a proxy for SES. The higher prevalence of anaemia amongst those who rode in a vehicle to school as against walking to school may corroborate the relationship between lack of physical exercise and the low‑grade inflammation that accompanies them and anaemia.

Public health interventions are known to work in reducing the prevalence of anaemia even though adherence is reported to be poor in most settings [40]. The WHO recommends such interventions as: improved dietary intake of food rich in iron in a bio‑available form, food fortification for high‑risk groups such as children under 5 years old and iron/folate supplementation for high‑risk children and adolescents [41]. Foods rich in dietary iron include lean meat and seafood. Non‑haeme dietary sources of iron include nuts, beans and vegetables. Because these foods may be expensive and out of reach of the poor, iron supplementation is advocated. Iron supplementation reduces anaemia in children, adolescents and pregnant women. As much as 42% and 55–70% of anaemia, and severe anaemia, respectively, in children is amenable to iron supplementation [42]. Indeed, iron supplementation has been reported (from randomised intervention studies) to increase haemoglobin concentrations in anaemic individuals by an average of 8–64 g/L for non‑pregnant women and 80 g/L for children [43]. These interventions need to be sustained in Ebonyi State and throughout Nigeria. However, it is important to review the messages and approaches used in such interventions as the data from this study shows that boys and the urban rich are affected more by severe anaemia than girls and rural dwellers, respectively. Additionally, the data suggest that older adolescents should be deliberately targeted with the once a week iron/folate supplementation recommended by the WHO to address the moderate public health importance of moderate and mild anaemia that is quite prevalent in that age‑bracket.

### Limitations

First, this study is limited by the small sample size and the enrolment of only school‑going adolescents. However, given the logistical challenges inherent in working in an environment where blood is regarded as life, it was difficult to get more subjects and their parents/guardians to consent to a finger prick. This is even more so when no honoraria were paid to participants. We nonetheless believe that the sample size is sufficient to draw attention to the wider implications of the current public health interventions aimed at reducing anaemia in children and adolescents. Second, school fees were used as proxy for socioeconomic status, instead of direct determination of the socioeconomic status of the families. Apparently, some middle‑class parents may send their children to schools where no fees are paid or to those that charge very high fees, depending on the premium they place on the education of their children and their assessment of what quality education truly means. This may have led to a wrong characterisation of the SES of some subjects. We, however, feel that the errors that could have resulted from this would be marginal and may not affect the overall conclusions from the data. We strongly feel that the data presented suffice to reach reasonable conclusions for a cross‑sectional study such as this. Our robust analysis and disaggregation of the data into SES groups and our significant findings and conclusions are important strengths of this study.

## Conclusion

The prevalence and dynamics of anaemia in children and adolescents of different residential and socio‑economic status groups in Ebonyi State, Nigeria was studied. Aggregate or total anaemia was found in 50.0% of the studied population (43.9% in males and 53.5% in females). Severe anaemia was present in 7.2% of the population (9.1% males and 6.1% females). Moderate and mild anaemia were found in 22.4% and 20.4% of the population, respectively, with a clear female preponderance. Aggregate or total anaemia was most prevalent in the urban upper SES group. Severe anaemia was most prevalent (13.0%) in the urban upper SES group. Moderate anaemia was most prevalent in the urban middle and upper SES groups (29.0% and 26.4%, respectively). Contrary to popular thought, severe anaemia was more prevalent in boys than girls and affected the urban higher SES group more than any other group. This calls for a rethink of the public health interventions used in Nigeria with a view to including adolescent boys in iron and folate supplementation and targeting urban dwellers of all SES groups.
